# Dynamics and origin of rebound viremia in SHIV-infected infant macaques following interruption of long-term ART

**DOI:** 10.1172/jci.insight.152526

**Published:** 2021-12-08

**Authors:** Veronica Obregon-Perko, Katherine M. Bricker, Gloria Mensah, Ferzan Uddin, Laura Rotolo, Daryll Vanover, Yesha Desai, Philip J. Santangelo, Sherrie Jean, Jennifer S. Wood, Fawn C. Connor-Stroud, Stephanie Ehnert, Stella J. Berendam, Shan Liang, Thomas H. Vanderford, Katharine J. Bar, George M. Shaw, Guido Silvestri, Amit Kumar, Genevieve G. Fouda, Sallie R. Permar, Ann Chahroudi

**Affiliations:** 1 Department of Pediatrics, Emory University School of Medicine, Atlanta, Georgia, USA.; 2 Department of Biomedical Engineering, Georgia Institute of Technology, Atlanta, Georgia, USA.; 3 Duke Human Vaccine Institute, Duke University Medical Center, Durham, North Carolina, USA.; 4 Yerkes National Primate Research Center, Emory University, Atlanta, Georgia, USA.; 5 Perelman School of Medicine, University of Pennsylvania, Philadelphia, Pennsylvania, USA.; 6 Center for Childhood Infections and Vaccines of Children’s Healthcare of Atlanta and Emory University, Atlanta, Georgia, USA.

**Keywords:** AIDS/HIV, Virology, AIDS vaccine

## Abstract

Understanding viral rebound in pediatric HIV-1 infection may inform the development of alternatives to lifelong antiretroviral therapy (ART) to achieve viral remission. We thus investigated viral rebound after analytical treatment interruption (ATI) in 10 infant macaques orally infected with SHIV.C.CH505 and treated with long-term ART. Rebound viremia was detected within 7 to 35 days of ATI in 9 of 10 animals, with posttreatment control of viremia seen in 5 of 5 Mamu-A*01^+^ macaques. Single-genome sequencing revealed that initial rebound virus was similar to viral DNA present in CD4^+^ T cells from blood, rectum, and lymph nodes before ATI. We assessed the earliest sites of viral reactivation immediately following ATI using ImmunoPET imaging. The largest increase in signal that preceded detectable viral RNA in plasma was found in the gastrointestinal (GI) tract, a site with relatively high SHIV RNA/DNA ratios in CD4^+^ T cells before ATI. Thus, the GI tract may be an initial source of rebound virus, but as ATI progresses, viral reactivation in other tissues likely contributes to the composition of plasma virus. Our study provides potentially novel insight into the features of viral rebound in pediatric infection and highlights the application of a noninvasive technique to monitor areas of HIV-1 expression in children.

## Graphical abstract

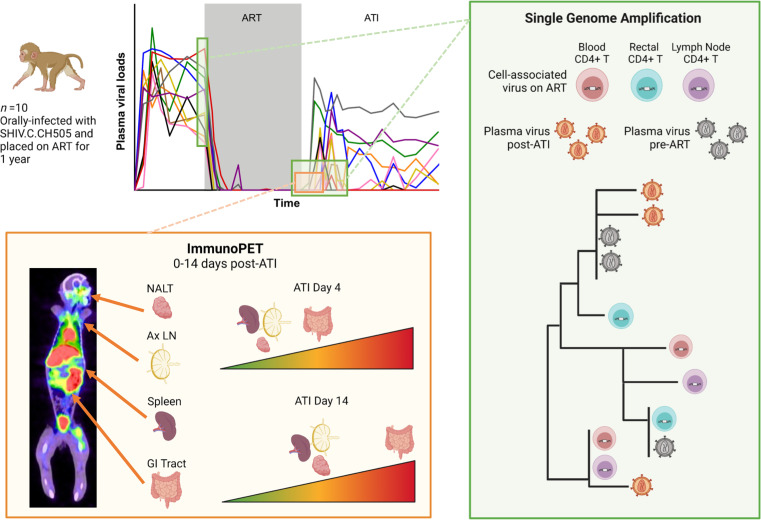

## Introduction

Early establishment of the long-lived viral reservoir, which persists on antiretroviral therapy (ART), remains a major obstacle for HIV-1 cure. Interruption of ART is typically followed by viral rebound and reestablishment of systemic infection, making adherence to ART a lifelong commitment. This is particularly daunting for the nearly 2 million children currently living with HIV-1 (1), who face a lifetime of daily medication that begins decades earlier than individuals infected in adulthood. Rare cases of sustained ART-free remission in children have been reported in instances in which ART was initiated early or very early after infection (2–4). However, blunting the size of the viral reservoir with early ART is challenging to implement in the setting of breastfeeding transmission, where diagnosis can be delayed. As such, there is a critical need to develop alternatives or complements to ART-based measures that can delay or prevent viral rebound in pediatric HIV-1 infection.

Understanding the dynamics and origin of viral rebound is a critical foundation for developing HIV-1 remission strategies. Studies of individuals with HIV-1 undergoing analytical treatment interruption (ATI) have shown that viral recrudescence typically occurs within 2 to 4 of weeks of ART cessation (5, 6), although this time frame can be longer for individuals treated early in infection. Several studies have reported multifocal viral reactivation from various tissue sites in the absence of ART, creating a polyclonal viral population in the plasma (7–9). Other reports show that viral rebound can also result from a small subset of clonally expanded cellular populations in the periphery (10). Viral recombination events and mutations that lead to escape from host immunity can further influence the composition of viral populations in plasma as ATI progresses (11–14). A few studies are available on the timing and clinical outcomes of viral rebound in children undergoing ART interruption (15–17), but detailed assessments of viral replication kinetics and the origin of viral rebound have yet to be addressed in this population. Viral recrudescence may be influenced by distinct features of pediatric immunity and HIV-1 infection (higher abundance of naive T cells, oral transmission route, high set point viral loads, etc.; ref. 18). Thus, deep investigations of viral rebound in this age group are needed to inform the development of pediatric-focused interventions for HIV-1 remission and cure.

Ethical considerations surrounding cessation of ART in children and the need for invasive sampling of tissues present challenges for thorough assessments of viral recrudescence. Nonhuman primate models of simian and simian/human immunodeficiency virus (SIV/SHIV) infection capture many aspects of HIV-1 pathogenesis and have proved highly valuable for preclinical research on virus persistence (19, 20). Our group has previously used orally infected infant rhesus macaques to evaluate sites of viral persistence and novel cure interventions (21–23). Here, we used a similar model of breastfeeding transmission and long-term ART to uncover the kinetics, composition, and anatomic origin of viral rebound in infant rhesus macaques following ATI. Using a combination of ImmunoPET imaging, single-genome analysis of plasma SHIV RNA and cell-associated SHIV DNA, and tissue-based virus quantification, we demonstrate that the gastrointestinal (GI) tract is a substantial site of viral RNA persistence that is capable of early SHIV.C.CH505 expansion in infant macaques following ATI, but, as ATI progresses, virus reactivation in other tissues likely contributes to rebound viremia. Our findings highlight viral rebound events in a relevant model of pediatric HIV-1 infection that will be valuable for informing the design of clinical trials in children to achieve HIV-1 remission.

## Results

### Viral rebound kinetics following ART interruption.

**Figure 1:**
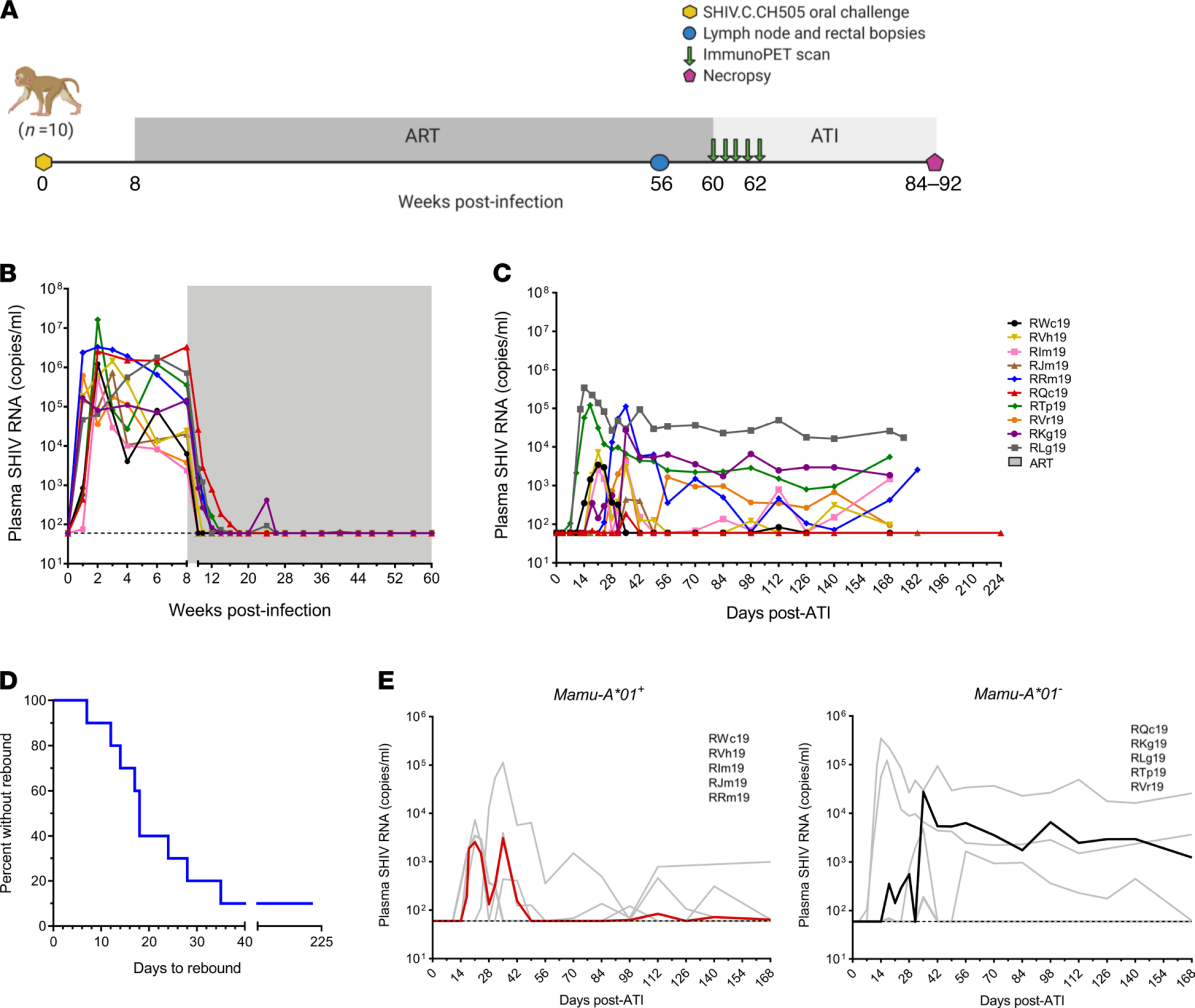
Kinetics of viral rebound following ART interruption in SHIV. C.CH505-infected infant rhesus macaques. (**A**) Study schematic showing timing of ART, tissue collections, and ImmunoPET imaging. (**B**) Plasma viral loads measured by real-time RT-PCR after infection and on long-term ART, indicated by shaded region (*n* = 10). (**C**) Plasma viral loads following analytical treatment interruption (ATI) (*n* = 10). For **B** and **C**, the dashed line reflects the LOD (60 copies/ml). (**D**) Kaplan-Meier curve of time to viral rebound (*n* = 10). (**E**) Viral replication kinetics during ATI in Mamu-A*01^+^ (left, *n* = 5) and Mamu-A*01^–^ (right, *n* = 5) macaques. Each curve represents 1 animal; bold lines represent the median. The study schematic was created with BioRender.com.

We first sought to evaluate the kinetics of viral rebound in 10 infant rhesus macaques orally infected with SHIV.C.CH505 and placed on daily ART at 8 weeks post-infection (wpi) (Figure 1A). Animals were maintained on ART for a minimum of 52 weeks and showed durable suppression of plasma viral loads for more than 32 weeks prior to ATI (Figure 1B). For this study, we designated the day of viral rebound as the time point with the first detectable plasma viral load (>60 copies/ml) that was followed by a subsequent detectable viral load. By this definition, viral rebound occurred 7–35 days after ATI in 9 of 10 animals (Figure 1D), with viral loads reaching pre-ART levels in most cases.

SHIV.C.CH505 replication kinetics during ATI were variable between animals (Figure 1C), but specific patterns were noted. All 5 animals positive for the Mamu-A*01 MHC I allele, known to be associated with CTL responses to an immunodominant Gag epitope (24), showed extended periods of posttreatment control (PTC), with plasma viral loads of less than 500 copies/ml for 8 weeks or more (Figure 1E). All animals displaying PTC during ATI also had viral loads that were, on average, 2 logs lower than in noncontrollers at time of ART initiation (Figure 1B). Viral rebound kinetics were less uniform in the 5 Mamu-A*01–negative animals. Sustained viremia with no periods of viral control following viral rebound was seen in 3 animals (RKg19, RLg19, RTp19). Finally, 1 animal (RQc19) had a single episode of detectable viremia at 35 days after ATI (186 copies/ml) but no viral rebound after 224 days of ATI (Figure 1, C and D). This was particularly striking considering that this animal had the highest level of viremia before ART (Figure 1B). Thus, while viral dynamics in plasma during ATI typically mirrored those seen prior to ART, viral loads before ART were not fully predictive of the observed patterns of viral rebound.

### Diversity and phylogeny of initial rebound virus.

**Figure 2:**
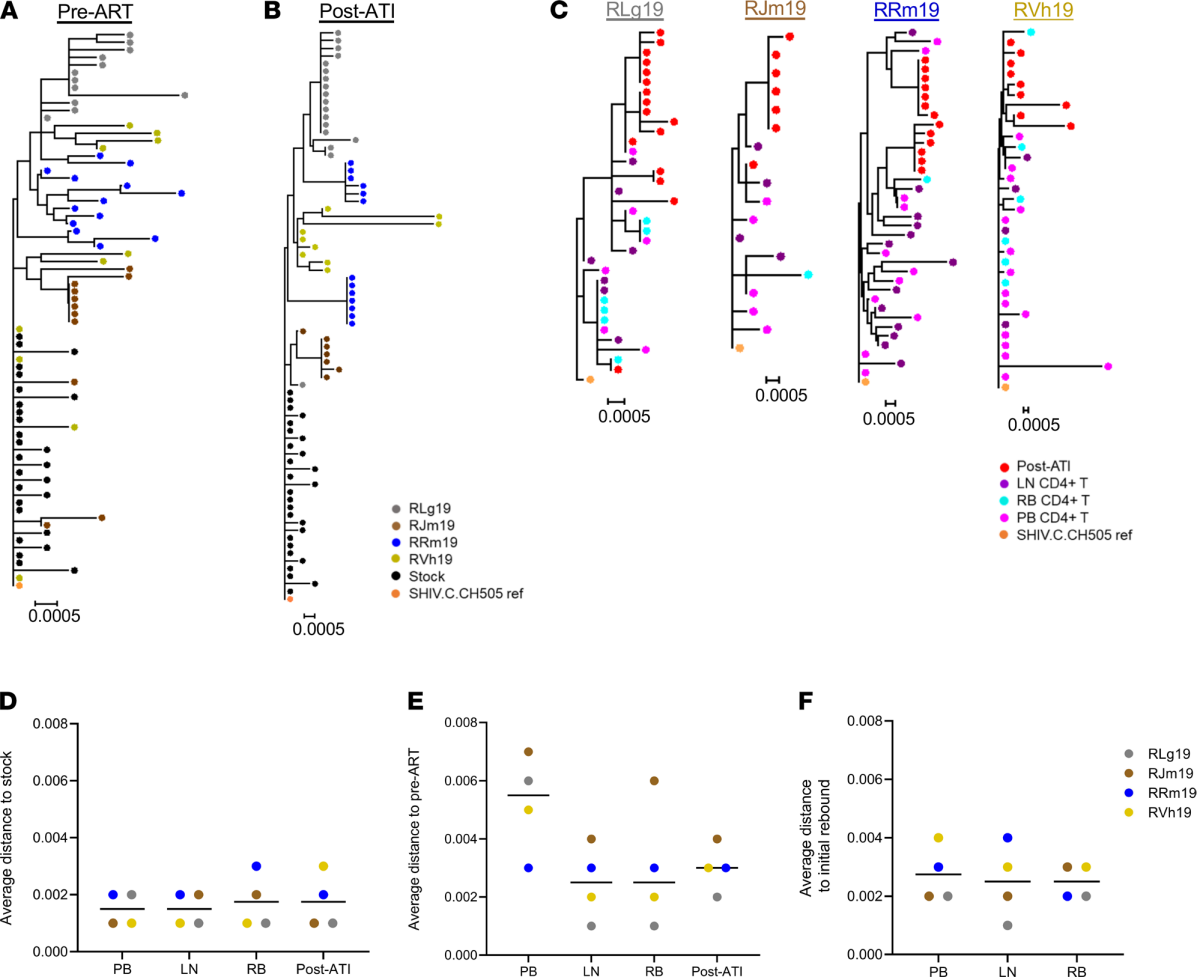
Phylogenetic analysis of env gene in plasma viral RNA sequences compared with viral DNA sequences in PB, LN, and RB. (**A** and **B**) Phylogenetic trees of viral *env* RNA sequences in plasma (**A**) prior to ART initiation (8 wpi) and (**B**) at initial viral rebound (1–5 weeks after ART cessation) (*n* = 4). (**C**) Phylogenetic trees of viral *env* RNA sequences in after ATI plasma with DNA sequences from PB, LN, and RB CD4^+^ T cells collected prior to ART interruption (>48 weeks on ART) (*n* = 4). (**D**–**F**) Average distance of *env* sequences to (**D**) challenge stock, (**E**) plasma before ART, and (**F**) initial rebound (post-ATI) plasma (*n* = 4). Lines represent the medians.

We next sought to characterize plasma and cell-associated SHIV.C.CH505 populations by single-genome amplification (SGA) of full-length *env*. Sequences were generated from a subset of 4 animals where amplicons could be obtained from all sites and time points of interest (Supplemental Table 1; supplemental material available online with this article; https://doi.org/10.1172/jci.insight.152526DS1). We first evaluated *env* diversity in viral RNA sequences isolated from plasma collected before ART and after ATI. Phylogenetic analysis of viral sequences in plasma at 8 wpi, just prior to ART initiation, revealed limited diversity in populations within each animal (Figure 2A). Divergence from the SHIV.C.CH505 challenge stock was also generally low. In animals RJm19 and RVh19, 3 of 11 (27%) and 4 of 10 (40%) *env* amplicons, respectively, clustered closest with sequences in the challenge stock and reference genome. Lower diversity and divergence in viral populations before ART, relative to previous reports for SIV and HIV-1 (25, 26), is likely attributable to the shorter duration of untreated infection or the use of a clonal virus stock in this study. Sequences after ATI in initial rebound virus, isolated 1–5 weeks after interruption and at the first viral load of more than 400 copies/ml, also did not show substantial diversity or divergence (Figure 2, B and D), here reflecting both the early initiation of ART and the early timing of sampling during rebound. It is anticipated that increased virus evolution would be found in later ATI time points with a longer period of viral replication, although this was not formally evaluated here.

Plasma *env* sequences were then aligned with viral *env* DNA sequences in CD4^+^ T cells collected just prior to ART cessation (Figure 2C). The degree of divergence from the SHIV.C.CH505 challenge stock sequence was equivalent among peripheral blood (PB), lymph node (LN), and rectal biopsy (RB) CD4^+^ T cells and initial plasma SHIV.C.CH505 RNA after ATI (Figure 2D). Similar observations were seen when comparing median distances to *env* sequences before ART (Figure 2E), although divergence in PB cells was slightly higher relative to LN and RB cells (PB vs. LN, *P* = 0.2399; PB vs. RB, *P* = 0.6021; LN vs. RB, *P* > 0.999). These findings are consistent with those of previous reports of limited viral evolution on long-term suppressive ART (27, 28). We evaluated whether initial rebound populations could be genetically related to viral DNA sequences in CD4^+^ T cells from blood and tissues on ART. With slight variability between animals, early post-ATI sequences in plasma clustered with those from PB, LN, and RB collected prior ART cessation (Figure 2C). Animals RLg19 and RJm19 had post-ATI plasma SHIV.C.CH505 *env* RNA sequences that were highly similar (>99.9%) to *env* DNA in CD4^+^ T cells from LN and PB, while RVh19, RRm19, and RLg19 also had initial rebound SHIV.C.CH505 RNA sequences that were more than 99.7% similar to DNA sequences from RB CD4^+^ T cells, with 1 plasma *env* RNA sequence identical to a RB sequence in RLg19 (Figure 2C). Nevertheless, the median average distance to post-ATI plasma sequences for all animals was comparable among PB, LN, and RB (Figure 2F), suggesting that *env* sequences present in each of these compartments prior to interruption of suppressive ART may represent viral variants that contribute to the composition of rebound virus in the plasma.

### Early anatomical sites of viral reactivation.

Our findings from single-genome sequencing, as well as those from similar studies of HIV-1 and SIV infection of adult humans and macaques, respectively, support the notion that viral reactivation following ART interruption is multifocal in nature, whereby reservoirs from multiple anatomical sources likely populate virus in the plasma (8, 9, 26). What remains less clear is the timing of viral reactivation in tissues and whether it occurs in synchrony between sites. To address this question, we used ImmunoPET (29) to visualize whole-body SHIV.C.CH505 Env expression before and after ATI in infant macaques by probing with radiolabeled gp120 V2 apex-binding PGT145 monoclonal antibody.

A series of control scans in a different set of SHIV.C.CH505-infected ART-treated infant macaques were performed (*n* = 4; 22). These animals were scanned with ^68^Ga-labeled PGT145 F(ab) and ^68^Ga-labeled isotype control IgG F(ab) to assess PGT145 and ^68^Ga background signal, respectively, under ART-mediated suppression of plasma viral loads. Images from scans after infusion with the ^68^Ga-IgG F(ab) showed the brightest signal in the kidneys and liver due to the accumulation of the probe at these sites after clearance from the periphery (Supplemental Figure 1A and refs. 29–31). All animals showed little-to-no ^68^Ga-IgG F(ab) signal throughout the rest of the body, although a moderate signal was noted in the hearts of 2 animals. Signals of similar magnitude to ^68^Ga-IgG F(ab) were also seen in the kidneys and liver after infusion with ^68^Ga-PGT145 F(ab) (Supplemental Figure 1B), and these tissues were not considered in further analyses of experimental animals. However, regions of low-to-moderate ^68^Ga-PGT145 signal were also seen in the nasal-associated lymphoid tissue (NALT) and GI tract, with a median total standard uptake value (SUV_total_) of 14 and 21 across each tissue, respectively, possibly attributable to viral protein debris or low-level viral protein expression on ART.

**Figure 3:**
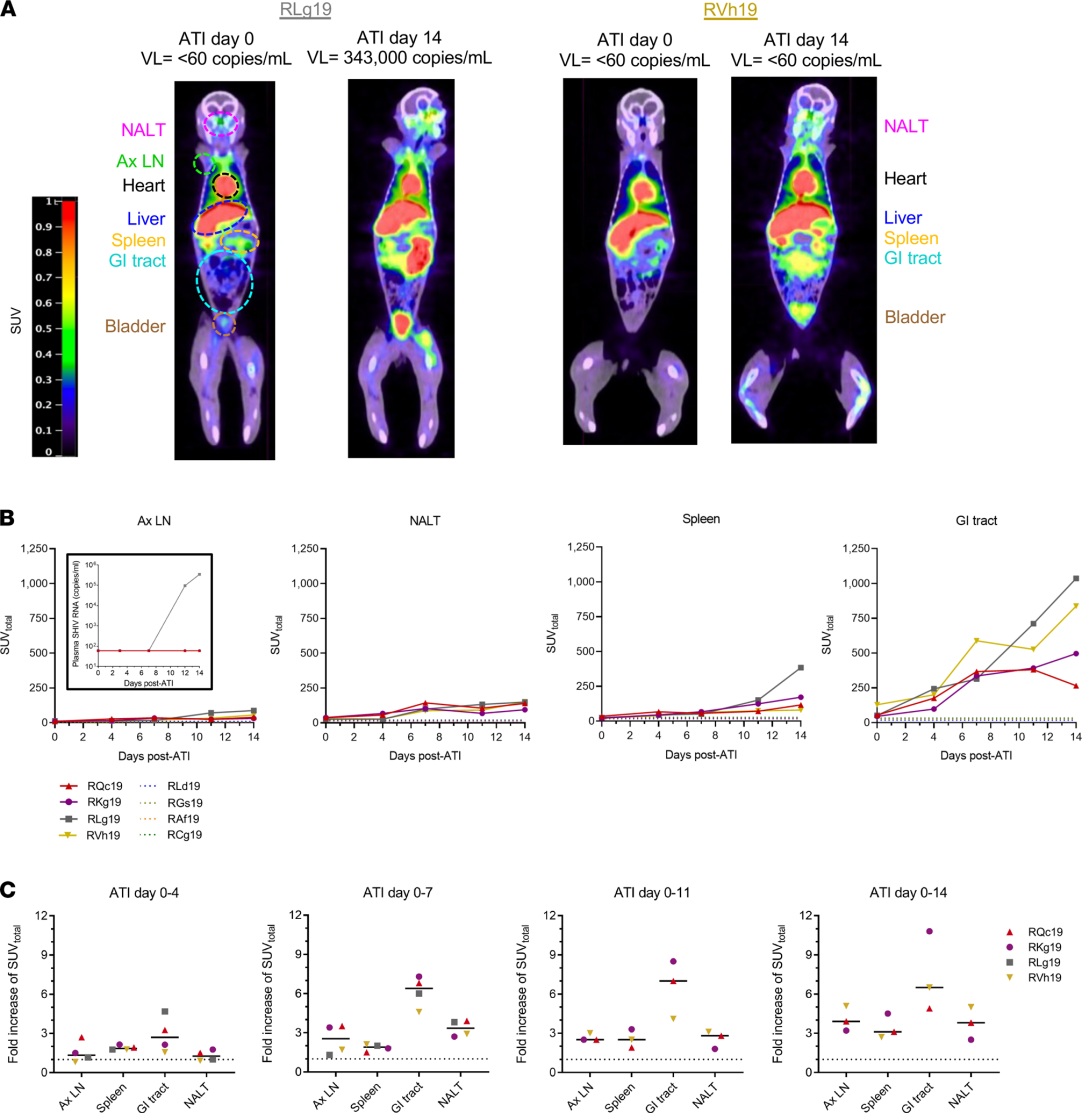
ImmunoPET to assess whole-body viral reactivation following ART interruption. (**A**) Standard uptake value (SUV) maps from 2 SHIV.C.CH505-infected infant rhesus macaques imaged with ^68^Ga-PGT145 F(ab). Representative images show frontal views from scans performed at 0 and 14 days after ATI. Images are from a single plane; all organs may not be visible in the same view. Plasma viral loads (VL) at the time of each scan are indicated above images. (**B**) Longitudinal quantification of SUV_total_ across anatomical sites during ATI (*n* = 4). Dashed lines represent values from a distinct group of ART-suppressed infant macaques scanned once after more than 32 weeks on ART without interruption (*n* = 4). The inset graph shows plasma viral loads at the time of each scan. (**C**) Fold increase in SUV_total_ at each anatomical site that preceded detectable plasma viremia from ATI day 0 to day 7 (*n* = 4) or to day 14 (*n* = 3). Lines represent the medians. NALT, nasal-associated lymphoid tissue; Ax LN, axillary lymph node; GI tract, gastrointestinal tract.

We next scanned the 4 experimental animals with the ^68^Ga-PGT145 probe to evaluate early sites of viral expansion in tissues following ART cessation. Scans were done once on long-term ART (>52 weeks on ART) and twice weekly for the 2 weeks immediately following ATI, for a total of 5 scans (Figure 1A). A notable increase in ^68^Ga-PGT145 signal was found in various tissues on the final scan (ATI day 14) relative to prior to ART interruption (ATI day 0; Figure 3A). Sites with a prominent signal on ATI day 14 included the NALT, axillary LNs, spleen, and GI tract. Although 1 animal was viremic by ATI day 14 (RLg19), increased probe signal at these tissue sites was still evident in animals that had undetectable plasma viral loads at the time of the final scan (RVh19, RKg19, RQc19; Figure 3A and Supplemental Figure 2), highlighting that SHIV.C.CH505 expansion in tissues can be visualized prior to evidence of viral rebound in plasma.

Images from all scans were quantified by calculating the ^68^Ga-PGT145 SUV_total_ for each tissue. Longitudinal analysis showed that ^68^Ga-PGT145 SUV_total_ values were typically elevated in all anatomical sites, with increased time after ART interruption, but the highest values were seen in the GI tract (Figure 3B). We considered the possibility that relatively higher SUV_total_ in the GI tract could be attributed to differences in organ size and probe uptake rather than reflective of a higher frequency of virally infected cells. Thus, we normalized the signal across tissues by calculating the fold change in ^68^Ga-PGT145 SUV_total_ from ATI day 0 to each of the subsequent scans and asked which site(s) showed the greatest elevation in signal prior to viral loads reaching detection in the plasma. Using this approach, we demonstrated that the GI tract had the largest median fold increase of SUV_total_ at the first scan and at all later points that preceded plasma viremia (Figure 3C). Earlier viral expansion in the GI tract was most prominent at ATI day 7, where the median signal increased from ATI day 0 by 6-fold but only by 2-fold in axillary LNs and spleen and 3-fold in NALT. The greatest fold change was observed in RLg19 at all sites at days 11 and 14, at which time the animal was viremic (Figure 3B). Interestingly, a ^68^Ga-PGT145 SUV_total_ increase in tissues was also noted in the 1 animal, RQc19, that did not have evidence of viral rebound by our definition (only a transient episode of detectable viremia occuring at 3 weeks after the final scan). Uniquely, however, the SUV_total_ declined between day 11 and 14 in the GI tract of RQc19, suggesting that this animal may have controlled emerging virus through immune-mediated mechanisms.

**Figure 4:**
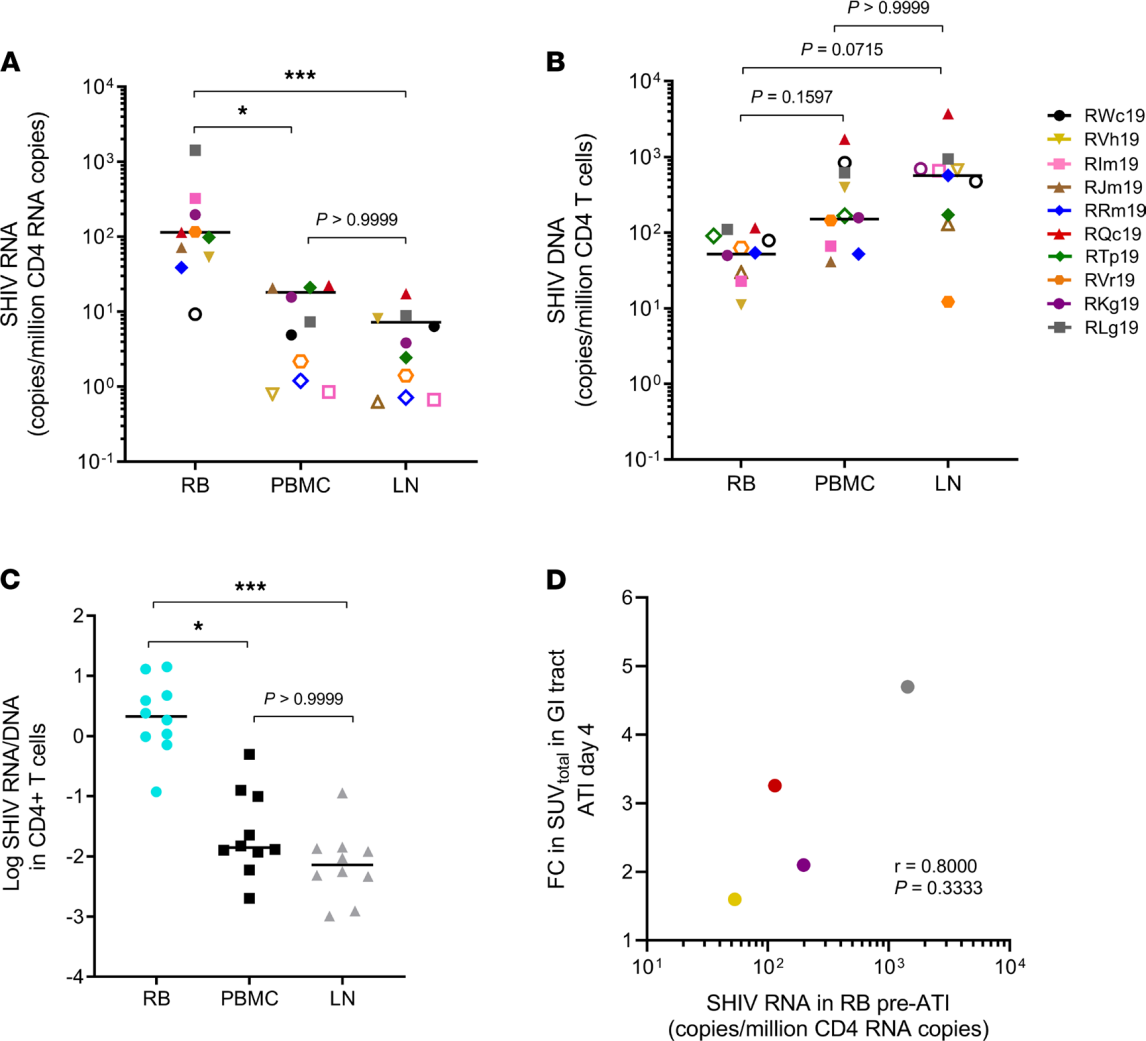
SHIV. C.CH505 DNA and RNA persistence in CD4^+^ T cells prior to ART interruption. (**A**) Cell-associated SHIV RNA and (**B**) SHIV DNA in enriched CD4^+^ T cells 4 weeks before ATI (48 weeks on ART). Open symbols indicate undetectable values, with LOD set based on cell input; these values were not included in calculations for the median or statistical significance. Kruskal-Wallis test with Dunn’s correction for multiple comparisons was used for statistics. (**C**) Log-transformed RNA/DNA ratios in CD4^+^ T cells from indicated sites. Statistical significance was determined by Friedman’s test with Dunn’s correction for multiple comparisons. In **A**–**C**, each point represents 1 animal (*n* = 10); lines represent the median. (**D**) Association between SHIV RNA in RB CD4^+^ T cells before ATI and the fold change (FC) in ImmunoPET signal from 0 to 4 days after ATI (*n* = 4). Spearman’s correlation was used for statistical analysis. *P < 0.05, **P < 0.01, ***P < 0.001.

Our group recently reported the GI tract as an active site of virus transcription during ART-mediated suppression in a separate, but similarly treated, group of SHIV.C.CH505-infected infant macaques (*n* = 6; ref. 22). Thus, we asked if enrichment of cell-associated viral RNA could explain why the GI tract is an early site of viral reactivation in this larger cohort of infant macaques (*n* = 10). Consistent with our previous findings, we found significantly higher viral RNA in CD4^+^ T cells from RB compared with CD4^+^ T cells from either PB (*P* = 0.02) or LN (*P* = 0.0006) (Figure 4A). Elevated RNA in the rectal compartment was not attributed to a higher frequency of infected cells, as determined by total viral DNA (Figure 4B). Viral RNA/DNA ratios were thus significantly higher in CD4^+^ T cells from RB compared with PB (*P* = 0.0110) and LN (*P* = 0.0004) (Figure 4C). We also observed an association between RB CD4^+^ T cell–associated SHIV.C.CH505 RNA and the magnitude of viral expansion in the GI tract (r = 0.8000, *P* = 0.3333). The animal with the highest viral RNA burden in the rectum before ATI (RLg19) had the greatest increase in ^68^Ga-PGT145 SUV_total_ signal at 4 days after ART interruption (Figure 4D). The converse was true for the animal with the lowest rectal viral RNA burden (RVh19). Taken together, these findings from ImmunoPET suggest that while rebound viremia is likely multifocal in origin, the GI tract is a major anatomical site of viral RNA persistence that is capable of early and substantial SHIV.C.CH505 expansion in infant macaques following ATI.

## Discussion

Greater knowledge of the kinetics and origin of viral rebound after ART cessation is critically needed to develop and evaluate HIV-1 cure strategies. Investigations in this area have largely focused on adults, contributing to a general lack of information on viral recrudescence in children perinatally infected with HIV-1. To address this knowledge gap, we used an established infant rhesus macaque model of SHIV.C.CH505 infection, whereby animals are orally challenged at 4 weeks of age to simulate infection in the perinatal period through breastfeeding (22), an understudied yet predominant transmission route in pediatric HIV-1. We provide evidence to support that the rebound plasma SHIV.C.CH505 RNA that follows interruption of long-term ART in infant macaques is multifocal in origin, with the GI tract showing the earliest evidence of virus reactivation, in keeping with the persistent viral transcription observed at this anatomic site during ART-mediated suppression of viremia.

In our model, plasma viral rebound was seen in all but 1 animal within 7–35 days of ART interruption, with a median time to rebound of 18 days. The CHER trial reported a median time to viral rebound, defined as viral loads of more than 400 copies/ml, of 1.8 months (approximately 50 days) in infants who initiated ART at under 12 weeks of age and received therapy for either 40 weeks or 96 weeks (32). Using the definition established in the CHER trial, the median time to rebound in our study would be 25 days. We measured plasma viral loads twice weekly immediately following ART interruption to more closely identify the exact time to viral rebound and so created our definition based on when viral loads reached detection (>60 copies/ml) rather than an established threshold. Differences between studies in sample size, viruses, timing of ART start, duration of ART, and antiretroviral drugs as well as our use of an animal model could explain the relatively shorter time to viral rebound seen here relative to the CHER study. Nevertheless, the heterogeneity seen in time to viral rebound make the infant macaque/SHIV model suitable for uncovering factors that extend viral remission.

The variable patterns of viral rebound that we observed provided insight into possible predictors of viral rebound events. Three animals with high viral loads at time of ART initiation (>100,000 copies/ml) had robust rebound viral loads that reached levels seen before ART and were sustained through 6 months of ATI. Approximately 1.5-log lower pre-ART viral loads and PTC of viral rebound were universal across animals expressing the Mamu-A*01 MHC I allele, highlighting a significant role for anti-Gag CTL responses in controlling SHIV.C.CH505 replication in infants, as has been described in other settings (24). Indeed, strong CTL responses and the expression of certain HLA alleles are commonly reported features in HIV-1 elite controllers who spontaneously maintain viral suppression without ART (33). Perhaps the most remarkable observation was the lack of viral rebound in 1 animal (RQc19) after 7 months of ATI. This was unexpected considering its high viral burden, both in the plasma prior to ART and within CD4^+^ T cells after long-term ART. Interestingly, ImmunoPET scans in RQc19 showed initial expansion of viral signal across various tissues that plateaued or declined, most pronounced in the GI tract, where the signal in the other 3 animals continued to increase over time (Figure 3). We hypothesize that viral reactivation occurred in this animal early in ATI, but systemic dissemination was ultimately blunted by immune-mediated mechanisms (34). Thus, while viral kinetics before ART and MHC I genotype could be partially predictive of ATI outcome, our observations in the nonrebounding animal underscore the importance of fully elucidating the factors that influence viral rebound events. Identifying strong viral and immune correlates of viral rebound will be critical in this area and is a topic of ongoing investigation by our group using a larger cohort of animals.

In addition to describing viral rebound kinetics, this work also proved informative regarding the anatomic origin of plasma rebound virus in SHIV.C.CH505-infected infant macaques after interruption of long-term ART. Phylogenetic analysis demonstrated that *env* sequences present in the plasma near the time of first detectable rebound closely matched those in SHIV.C.CH505 DNA isolated from PB, LN, and RB CD4^+^ T cells prior to ART interruption, suggesting a possible multifocal contribution to rebound viremia. ImmunoPET scans to visualize whole-body SHIV.C.CH505 Env expression confirmed viral reactivation after ART interruption at multiple sites, including the NALT, axillary LN, spleen, and GI tract. However, the magnitude and timing of viral reactivation was variable between sites, with the fastest and most pronounced rise in viral signal in the GI tract. The persistently elevated SHIV.C.CH505 RNA in the GI tract we observed during ART could underly the early expansion of virally infected cells in this region and would align with a previous report of transcriptionally active cells contributing to HIV-1 rebound (10). Data from adult macaques have also implicated lymphoid tissue, including from the GI tract, as a persistent source of viral RNA during suppressive ART (35, 36). Future studies using barcoded viruses may provide increased precision regarding viral reactivation rates from various tissues and cell types as well as recombination events that may contribute to rebound viremia (26, 37, 38). Taken together, the viral sequencing and ImmunoPET imaging results shown here support the idea that viral rebound in SHIV-infected infant macaques is not attributed to expansion from a single tissue but is likely a consequence viral reactivation at multiple anatomic locations. While multifocal viral rebound has been described in adults (7–9, 26), this is, to our knowledge, the first study to explore the origin of rebound viremia in a pediatric model of HIV-1.

This study has several limitations. First, we note that the phylogenetic analysis was restricted to 4 animals. The small size of the infant macaques made it difficult to obtain sufficient cell numbers from lymphoid and rectal tissues for SGA across all 10 animals. Related to this was the low number of amplicons obtained from these sites relative to plasma and PB CD4^+^ T cells. Visualization of viral reactivation by ImmunoPET was also restricted to a subset of 4 animals based on cost and other practical considerations. We were unable to explore the central nervous system as a contributor to viral rebound, as the probe we used for ImmunoPET does not cross the blood brain barrier. However, we have previously shown that SHIV.C.CH505 DNA in the brain is largely undetectable in infant macaques after 1 year of ART (22), making it unlikely that the CNS is a major source of rebound viremia in this model. The ImmunoPET imaging detects cells and tissues that express Env, recognized by PGT145. We recognize that this methodology is unlikely to detect every reservoir cell in the body. It is also possible that some of the signal in tissues after ATI reflects Env-expressing cells in the blood vessels traversing that tissue. However, we feel this is unlikely to represent the majority of the ImmunoPET signal, as we did not see an elevation in plasma viral loads or in signal in the heart, a highly vascular organ, that coincided with rises in signal at other tissue sites. Further, if ImmunoPET signal was largely attributed to blood, one might expect proportional rises in signal across all tissue sites, which we did not observe. The early and synchronized timing of ART initiation, as well as the use of a transmitted/founder SHIV, may have contributed to a lower heterogeneity in time to rebound than has been seen in human clinical trials. Larger cohort sizes and perhaps staggered ART timing may be necessary to provide sufficient statistical power for future studies exploring correlates of viral rebound in this model. While the use of a SHIV provides an opportunity to directly evaluate anti-HIV envelope targeting cure strategies, SHIV models also carry the recognized limitation of lower viral loads compared with SIV. Thus, confirming that our findings in a model of SIV infection could be of value to this area of research.

In summary, we have used SHIV-infected infant rhesus macaques to evaluate the kinetics and origin of viral rebound after discontinuation of long-term ART. We describe variable patterns of viral replication during ATI that did not always mirror those seen in the acute phase of infection. While rebound virus in the weeks following ATI closely matched viral DNA in blood, LNs, and the GI tract, the latter was the earliest and greatest site of viral expansion throughout the first 14 days. While it will be important to validate our findings from infant macaques in children with HIV-1, the longitudinal application of these innovative techniques in a relevant animal model is a significant step forward in the advancement of understanding HIV-1 rebound in perinatal infection. Furthermore, we demonstrate the feasibility of utilizing an imaging technique in children undergoing ATI or treatment with a potentially novel cure intervention to monitor HIV-1 reactivation in areas that would be otherwise inaccessible or would require more invasive strategies to sample in a clinical setting. This work may inform the rational design of trials involving ART interruption in children.

## Methods

### Animals and infection.

Ten infant Indian rhesus macaques (*Macaca mulatta*), with exclusion of Mamu-B*08^+^ and Mamu-B*17^+^ animals, were enrolled in this study. The cohort consisted of 5 female and 5 male animals. The animals were born at the Yerkes National Primate Research Center (YNPRC) to dams housed in indoor/outdoor group housing. The infants were removed from the dams when they were approximately 2 weeks old and transferred to a nursery, where they were housed in social groups for the duration of the study. The infants were fed in accordance with the YNPRC standard operating procedures for nonhuman primate feeding. After being removed from the dam, infants were fed center-approved milk replacer (Similac Advance, OptiGro Infant Formula with Iron and/or Similac Soy Isomil OptiGro Infant Formula with Iron; Abbott Nutrition) until 14 weeks of age. Infants were provided softened standard primate jumbo chow biscuits (Jumbo Monkey Diet 5037; Purina Mills) and a portion of fruit starting around 4 weeks of age. As animals aged, additional enrichment was provided daily, in the form of various fresh produce.

All animals were infected with SHIV.C.CH505.375H.dCT, as previously described (22). In brief, animals were challenged at 4 weeks of age by oral administration in 2 consecutive doses spaced 24 hours apart. Starting challenge dose was 45 ng p27 Ag (1.6 × 10^8^ vRNA molecules, 8.0 × 10^7^ virions). If plasma viremia was not detected by 1 week after inoculation (>60 copies/ml), the challenge was repeated with an escalated dose. Infection was achieved with doses ranging from 45 ng to 360 ng p27 Ag.

### ART.

All animals proceeded to treatment with a potent 3-drug ART regimen at 8 weeks after infection. The formulation contained two reverse transcriptase inhibitors, 5.1 mg/kg tenofovir disoproxil fumarate and 40 mg/kg emtricitabine, plus 2.5 mg/kg of the integrase inhibitor dolutegravir. ART cocktail was administered subcutaneously once daily at 1 ml/kg. Animals were maintained on daily ART for a minimum of 52 weeks prior to ATI.

### Sample collection and processing.

Blood samples were collected regularly and used for complete blood counts, routine serum chemistries, and immunostaining. PBMCs were isolated by density gradient centrifugation. Axillary or inguinal LNs were collected throughout the study and mechanically disrupted over a 70 μm cell strainer to prepare a single-cell suspension. Rectal biopsies were also collected, enzymatically digested with collagenase and DNase I for 2 hours at 37°C, and then passed through a 70 μm cell strainer. All cell suspensions were washed and immediately used for downstream assays or cryopreserved in 10% DMSO-FBS until use.

### Plasma SHIV RNA and cell-associated DNA/RNA quantification.

Plasma viral quantification was performed as in previous studies (39, 40). Quantification of cell-associated viral DNA was done on enriched CD4^+^ T cells with published primer/probe sets (41). Frozen CD4^+^ T cell pellets (~100,000 cells) collected from blood, LN, and rectal biopsies were lysed in proteinase K and used directly in the PCR reaction. Lysates were then quantified for levels of SHIV *gag* DNA relative to cell equivalents, as determined by monkey albumin gene copies (42). For cell-associated viral RNA, all cell pellets (300,000 to 2 million cells) were lysed in RLT plus containing β-mercaptoethanol, purified for RNA, and reverse transcribed (High Capacity cDNA Reverse Transcription Kit, Thermo Fisher Scientific). SHIV *gag* RNA levels were quantified and normalized to host *CD4* RNA copy numbers as described elsewhere (41).

All host and viral targets were detected by TaqMan assay on an ABI 7500 system in duplicate. PCR conditions have been optimized to detect a minimum of 3 copies of viral DNA or cDNA per reaction, and so the limit of detection (LOD) for each sample was calculated to be 3 SHIV *gag* copies/number of host cell equivalents or host cell RNA copies detected in the same reaction. Samples below the LOD are indicated by an open symbol on data plots.

### SGA.

SGAs of envelope gene were obtained from the plasma and cells of infected animals as described previously (43). Briefly, viral RNA was isolated from plasma of each animal using the QiaAmp viral RNA mini kit. Cell-associated DNA was isolated from CD4^+^ cells obtained from LN, rectal biopsies, and PBMCs using the DNeasy kit (Qiagen), as per manufacturer’s instructions.

cDNA was generated using SuperScript III reverse transcriptase mix (Invitrogen) and antisense primer SHIVEnvR3-out 5′-CTAATTCCTGGTCCTGAGGTGTAATCCTG-3′located in the nef reading frame (nt 9250-9278 SHIV1157ipD3N4). The cDNA or DNA suspensions were diluted and PCR amplified in Platinum Taq DNA polymerase High Fidelity (Invitrogen), such that 25% of reactions were positive to maximize the likelihood of SGA. The first round of PCR amplification was conducted using SHIVEnvR3-out and SIVmac766.F4-out 5′-TCATATCTATAATAGACATGGAGACACCC-3′ as primers. The second round of PCR amplification was conducted using 2 μl first-round PCR product as template and SIVmac766.F2-in 5′-GGAAATCCTCTCTCAACTATACCGCCCTC-3′ and SIVmac766.R2-in 5′-CTATTGCCAATTTGTAACTCATTGTTC-3′ as primers. Amplification conditions for round 1 and round 2 PCR were 1 cycle of 94°C for 2 minutes, 35 cycles of 94°C for 15 seconds, 55°C for 30 seconds, and 68°C for 4 minutes, followed by 1 cycle of 68°C for 10 minutes. Round 2 PCR amplicons were visualized by agarose gel electrophoresis and directly sequenced using an ABI3730xl genetic analyzer (Applied Biosystems). Full sequences were constructed from overlapping sequences from each amplicon in Sequencher (Gene Codes Inc.). Sequences with 2 or more double peaks were discarded, as this indicates amplification from multiple templates. Sequences with 1 double peak were retained, with the double peak attributed to Taq polymerase error. Sequence alignments and phylogenetic trees were constructed using Seaview (44) and MEGA6 (45), respectively. All sequences have been deposited into Genbank (accession MZ852896-MZ853066).

### ImmunoPET.

A monoclonal antibody against HIV-1 gp120 (PGT145; ref. 46) (Catalent) and an isotype control IgG (Catalent) were used for in vivo imaging. Antibodies were first modified into F(ab) fragments using the papain-based Pierce F(ab) Preparation Kit (Thermo Fisher Scientific) according to the manufacturer’s instructions. F(ab) fragments were buffer exchanged into 1 mg doses in 0.1 M Sorensen’s Phosphate Buffer (Electron Microscopy Sciences) (pH 8.9) using 10 kDa Amicon Ultra spin filters (MilliporeSigma). For conjugation to the chelator, THP-NCS (CheMatech) was resuspended at 10 mg/mL in DMSO (MilliporeSigma) and added at a 2-fold molar excess to the F(ab) dose. Mixture was placed on a ThermoMixer (Eppendorf) at 40*g* at 37°C for 90 minutes. Unbound chelator was removed using 4 washes of 0.1 M phosphate buffer (pH 7.3) in 10 kDa Amicon Ultra spin filters. Doses were lyophilized overnight and stored at 4°C until use.

For ^68^Ga labeling, lyophilized F(ab)-THP was resuspended in chelexed 0.1 M NH_4_OAc, pH 5.5 (MilliporeSigma). ^68^Ga was neutralized by injecting 1 mL ammonium acetate into the vial. F(ab)-THP was then mixed with neutralized ^68^Ga at a ratio of approximately 5 mCi/mL and incubated at room temperature for 15 minutes. The antibody conjugates typically labeled in the range of 1.2 to 2.5 mCi/mL. Each dose was buffer exchanged with pharmaceutical-grade saline 3 times using a 10 kDa centrifugal filter to a final volume of about 75 μL. The conjugated antibody was then diluted to 4 mL pharmaceutical-grade sterile saline in a sterile syringe. Under sedation, animals were injected intravenously with 1 mg ^68^Ga-labeled PGT145 or control IgG F(ab) (0.5–1.0 mCi per dose) and scanned 4 hours later.

All animals were anesthetized and imaged using a Siemens Biograph 40 PET/CT, using image settings for ^68^Ga. Approximately 250–300 image slices were collected for each animal. PET/CT fusions were analyzed using MIM software version 6.8. The PET edge tool was used to create a 3D ROI around the kidneys in each scan. The 3D brush tool was used to ensure that the 3D ROI only surrounded the organ and did not include or exclude any tissue, as appropriate. SUVs were then measured within the organs.

### Statistics.

All data graphs were generated on Prism version 8 (GraphPad). To analyze changes in cell-associated SHIV.C.CH505 DNA or RNA levels, we used Kruskal-Wallis for unpaired samples and Friedman’s test for paired samples, with Dunn’s correction for multiple comparisons in both. Undetectable viral DNA and RNA values were excluded from statistics and calculations for the median, as the quantified LOD represents only an estimate of reservoir size based on cell input. Spearman’s correlation was used to assess the association between ImmunoPET signal and viral RNA burden. *P* ≤ 0.05 was considered statistically significant.

### Study approval.

All procedures were reviewed and approved by the Emory University and YNPRC Institutional Animal Care and Use Committee. All animals were cared for in accordance with applicable Emory University and YNPRC regulations. The YNPRC is AAALAC accredited.

## Author contributions

VOP wrote the manuscript, conducted experiments, curated data, and performed analysis with supervision from AC. VOP, KMB, GM, FU, LR, DV, YD, and SL conducted experiments. AK led SGA studies and analysis, with coordination and input from SJB. Supervision of virologic assays and ImmunoPET scans were done by THV and PJS, respectively. SJ, JSW, FCCS, and SE coordinated animal support and care. KJB and GMS provided resources. AC, GGF, SRP, and GS conceptualized studies and acquired funding. All authors contributed to data interpretation and manuscript editing.

## Supplementary Material

Supplemental data

## Version 1 10/26/2021

In-Press Preview

## Version 2 12/08/2021

Electronic publication
