# Supplementary material for: Dynamics and origin of rebound viremia in SHIV-infected infant macaques following interruption of long-term ART
Source: JCI Insight. 2021 Dec 8;6(23):e152526. doi: 10.1172/jci.insight.152526 (PMC8675190; doi:10.1172/jci.insight.152526)
Supplement: Supplemental data [file jciinsight-6-152526-s005.pdf]

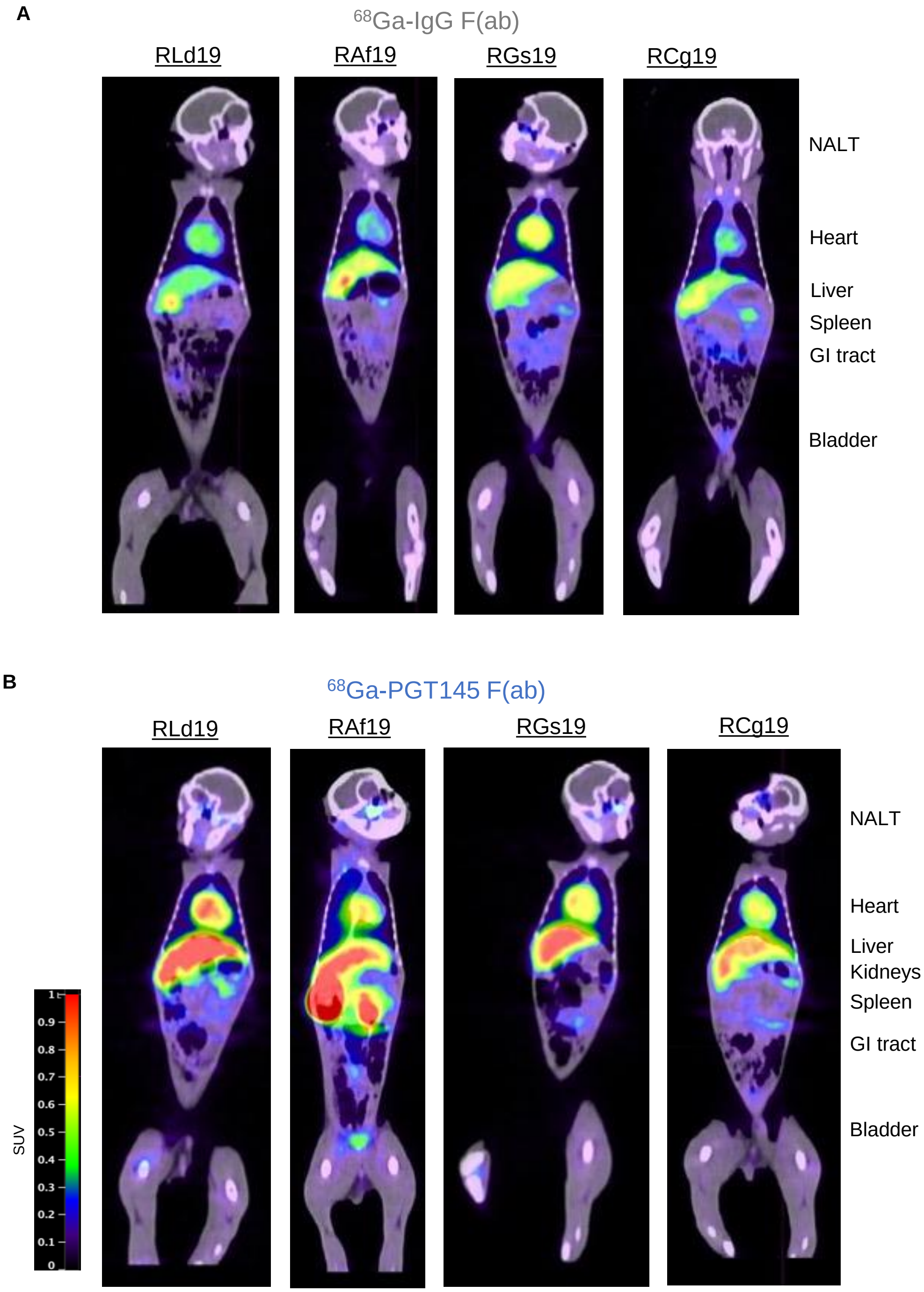

Supplemental Figure 1. ImmunoPET scans to assess ^{68}Ga and PGT145 background signal. Representative standard uptake value (SUV) maps from four SHIV.C.CH505-infected ART-suppressed infant rhesus macaques imaged with (A) ^{68}Ga-IgG F(ab) or (B) ^{68}Ga-PGT145 F(ab) probe after >32 weeks on ART without interruption. Images show frontal views. Images are from a single plane, all organs may not be visible in the same view. *NALT: nasal-associated lymphoid tissue; Ax LN: axillary lymph node; GI tract: gastrointestinal tract*

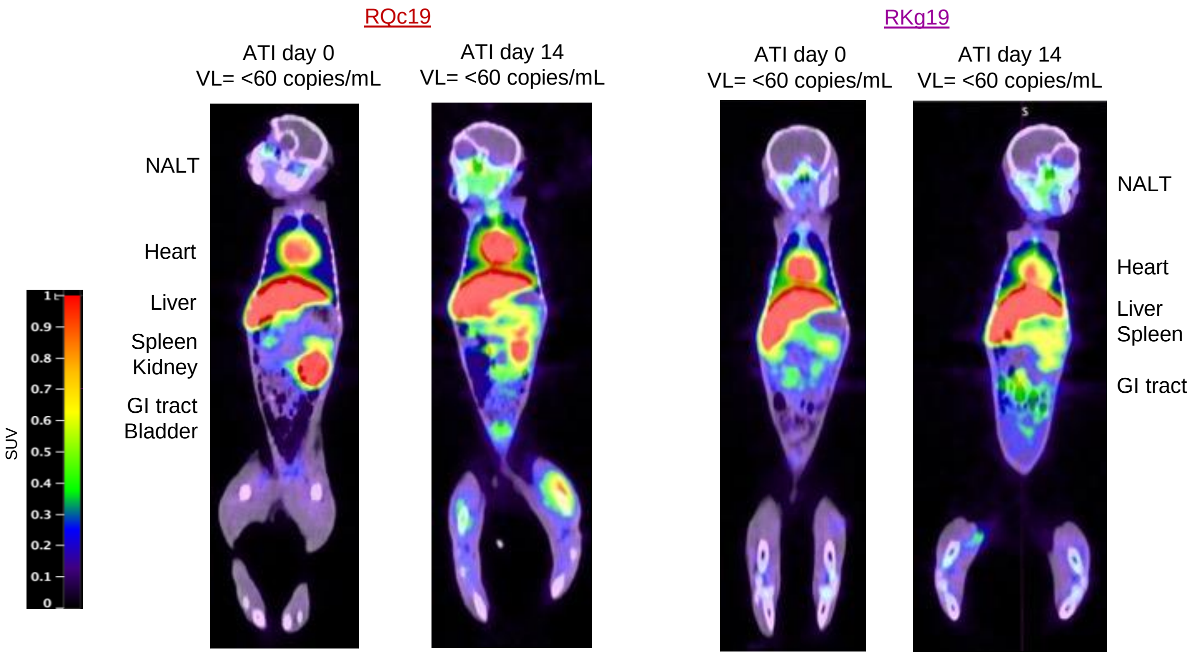

Supplemental Figure 2. ImmunoPET scans with ^{68}Ga-PGT145 F(ab) following ART interruption. Standard uptake value (SUV) maps from two SHIV.C.CH505-infected infant rhesus macaques imaged with ^{68}Ga-PGT145 F(ab). Representative images show frontal views from scans performed at 0 and 14 days post-ATI. Images are from a single plane, all organs may not be visible in the same view. Plasma viral loads (VL) at the time of each scan are indicated above images. *NALT: nasal-associated lymphoid tissue; Ax LN: axillary lymph node; GI tract: gastrointestinal tract*

Supplemental Table 1. Number of sequences obtained by SGA

Animal ID	Pre-ART Plasma	Post-ATI Plasma	RB CD4+ T	LN CD4+ T	PB CD4+ T
RLg19	12	17	6	7	9
RVh19	10	9	6	4	16
RJm19	11	7	1	4	6
RRm19	13	13	1	13	11
